# Supplementary material for: Correlation with Apoptosis Process through RNA-Seq Data Analysis of Hep3B Hepatocellular Carcinoma Cells Treated with Glehnia littoralis Extract (GLE)
Source: Int J Mol Sci. 2024 Aug 30;25(17):9462. doi: 10.3390/ijms25179462 (PMC11394729; doi:10.3390/ijms25179462)
Supplement: Supplementary file 1 [file ijms-25-09462-s001.zip › ijms-3110985 Supplementary.pdf]

Supplementary Table S1. Data used in the circular cluster heatmap to see the increase or decrease in Apoptotic process-related genes used in the PPI Network

**Gene Symbol**	**getC**	**getT**
APAF1	3.552	4.872
ATF4	8.771	9.897
ATG4D	3.077	4.098
ATG5	4.051	5.271
BAG3	4.261	6.424
BCL2L11	3.635	5.487
BCL7B	3.107	4.398
BCL7C	4.021	2.852
BIRC3	7.244	6.124
BNIP2	4.753	5.923
BOK	4.051	2.390
CCN1	3.077	4.527
CDK5	4.235	3.202
CHAC1	5.821	8.317
CSRNP1	3.464	4.900
CX3CL1	4.478	2.466
DDIT3	4.235	7.362
DDIT4	5.405	6.863
DEDD2	4.902	6.502
DHCR24	8.277	7.151
ENDOG	5.126	4.098
FADD	6.695	5.138
FEM1B	4.021	5.079
FOXO3	5.944	7.171
GADD45A	4.325	5.969
GADD45B	3.823	7.935
GADD45G	1.914	4.562
GAS2	4.455	3.013
GCLM	5.416	6.974
GPAM	4.124	2.735
GPER1	5.721	4.121
HMOX1	4.349	5.440
HNF1A	5.068	3.288
HSP90AA1	11.224	12.246
IER3	7.651	5.782
IKBKE	4.825	3.519

INHBB	4.478	3.408
JUN	7.426	9.105
KIFAP3	2.921	4.492
MAP1S	4.036	5.459
MARCKS	8.139	6.958
MEF2D	3.892	5.532
MINDY3	3.573	4.830
MSX1	3.841	5.091
NFKBIA	8.758	7.512
PEA15	5.405	6.429
PIM1	3.371	5.261
PPP1R15A	5.582	8.051
RAD9A	4.919	6.315
RHOA	8.251	7.104
RRAGC	5.633	7.207
RRN3	5.104	6.493
RTKN	6.379	4.900
SAV1	3.164	4.168
SEMA3A	6.345	5.313
SGK1	5.357	2.390
SIRT1	3.694	5.217
SOD2	8.467	7.291
SOX9	6.385	5.206
TCIRG1	5.623	4.190
TRIB3	5.623	6.780
VDAC2	5.835	6.852
ZC3H12A	6.889	4.966
ZFP36L1	8.155	6.994

Supplementary Table S2. Five genes with large increases and decreases before and after GLE treatment (Normalized data (log_2))

Increase Gene			Decrease Gene		
Gene Symbol	Control	Test	Gene Symbol	Control	Test
GADD45B	3.823	7.935	SGK1	5.357	2.390
DDIT3	4.235	7.362	CX3CL1	4.478	2.466
GADD45G	1.914	4.562	ZC3H12A	6.889	4.966
CHAC1	5.821	8.317	IER3	7.651	5.782
PPP1R15A	5.582	8.051	HNF1A	5.068	3.288

GEO number is GSE276192.
